# Therapeutic and tectonic keratoplasty with simple cryopreserved remnants of donor corneas: an 11 year retrospective case series

**DOI:** 10.1038/s41598-022-10994-3

**Published:** 2022-05-05

**Authors:** Jae-Gon Kim, Jong Hwa Jun

**Affiliations:** Department of Ophthalmology, Keimyung University School of Medicine, Dongsan Medical Center, Daegu, Korea

**Keywords:** Anatomy, Medical research

## Abstract

This study sought to describe the use of deep-frozen donor corneal remnants preserved after keratoplasty procedures for therapeutic or tectonic keratoplasty without subsequent optical keratoplasty. This single-center retrospective consecutive case series analyzed the electronic medical records of patients who had undergone therapeutic or tectonic keratoplasty using deep-frozen donor remains preserved in Optisol-GS, for the past 11 years at Keimyung University Dongsan Medical Center. Fifty-five surgical cases in 46 patients were included. Twenty-three surgical cases in 18 patients underwent therapeutic keratoplasty for refractory infectious corneal ulcer. Complete eradication of primary infection was achieved in 14 patients (77.8%). Tectonic keratoplasty were performed 32 cases in 28 patients. Twenty-seven of 28 patients were ultimately able to maintain anatomical integrity (96.4%). Mean uncorrected visual acuity improved from 1.77 ± 0.94 preoperatively to 1.31 ± 0.95 at the last follow-up postoperatively in the tectonic graft group by logarithm of the minimal angle of resolution (*P* = 0.002). There were no cases of graft rejection. Keratoplasty using cryopreserved donor tissue is a suitable surgical alternative for infectious or non-infectious corneal ulcers in elderly patients or patients with poor general condition. It could be a viable alternative to overcome the shortage of corneal donors.

## Introduction

Corneal deep ulceration and refractory infectious keratitis are emergent situations that require prompt surgical intervention. In these cases, the promptness of surgical intervention plays a crucial role in determining the prognosis of the condition. A variety of surgical options, ranging from corneal gluing, collagen crosslinking, amniotic membrane transplantation (AMT), conjunctival flap, and corneal transplantation have been utilized as therapeutic modalities for these conditions^1–3^. These interventions can be promptly implemented, except for those that require fresh corneal grafts. However, these techniques are intended not to restore vision but to temporarily manage the condition, with the goal of performing subsequent optical keratoplasty^3^. For AMT, the amniotic membrane could be lost before epithelialization is complete if the underlying disease is not properly controlled. In addition, AMT, scleral grafts, and conjunctival flaps cause cosmetic problems due to tissue opacity, and the post-transplant tissue absorption rate is higher than that of normal corneal tissue, which could compromise the stability of tissue integrity over time^4^.

However, corneal donation is drastically lower in East Asian countries compared with Western countries, leading to a grossly insufficient supply of donor corneas^5^. Furthermore, even if donor tissue can be obtained in a timely manner, therapeutic and tectonic keratoplasty procedures have higher rejection risks than conventional keratoplasty^6^. This is partly because the use of immunosuppressants to prevent graft rejection is contraindicated in the treatment of corneal infectious ulcers due to the risk of abrupt exacerbation of an already existing infection^7,8^.

Due to these limitations, donor corneal tissues that are not suitable for transplantation or remaining donor tissues after transplantation are cryopreserved and stored for later use^9^. Some studies have reported that corneal endothelial cells around the perforated site migrate to cover the posterior side of the cryopreserved donor graft after transplantation^10^. However, these tissues are primarily transplanted in the marginal region or used only as a temporary step prior to subsequent optical keratoplasty^11–14^.

This study aims to report the advantages of grafting corneal remnants cryopreserved with Optisol-GS for emergency tectonic or therapeutic keratoplasty. This technique can be used for treatment of corneal ulcers or perforations in patients with medical conditions that would contraindicate immunosuppression. This approach enables appropriate distribution of fresh cornea grafts to patients with high potential for visual improvement, while still maintaining a readily available source of cryopreserved corneal grafts for time-sensitive treatment of corneal perforation and infectious ulceration with therapeutic or tectonic keratoplasty. This prioritization is of utmost importance when donor tissue is limited, such as in East Asian countries.

## Methods

This study was approved by the Keimyung University Dongsan Hospital Institutional Review Board on December 11, 2020 (approval number: DSMC 2020-12-012). It was conducted in adherence to the tenets of the Declaration of Helsinki and followed all guidelines for experimental investigation in human subjects. This was a retrospective study of patients who underwent tectonic or therapeutic keratoplasty using cryopreserved cornea remnants at Keimyung University Dongsan Medical Center. This study is a retrospective study of medical records, and the Keimyung University Dongsan Hospital Institutional Review Board has determined that the risk to participants in the study does not exceed the minimum risk. Therefore, informed consent was waived and not required. In our institution, an average of 30 cases of corneal transplantation including penetrating keratoplasty (PKP) and descemet stripping automated endothelial keratoplasty (DSAEK) are performed annually, and the remaining donor cornea is cryopreserved in all cases. The study included 55 surgical interventions performed on 46 patients with impending/apparent corneal perforation or refractory infectious keratitis from January 2010 to May 2021. The collected data included age, gender, laterality of surgery, preoperative diagnosis, causative organism if a culture was conducted, interval from diagnosis to graft, previous ophthalmic and medical history, type of keratoplasty, pre- and postoperative uncorrected visual acuity (UCVA) as logarithm of the minimal angle of resolution (LogMAR), type of donor remnant tissue, graft size, postoperative follow-up duration, final graft status by slit-lamp examination, and prognosis. Patients were divided into therapeutic and tectonic groups according to the purpose of surgery. Therapeutic keratoplasty was performed primarily in patients with refractory infectious keratitis to eradicate the infection source^15^. Tectonic keratoplasty was performed in cases of impending or apparent corneal perforation due to insults such as trauma, sterile deep corneal ulcer, or keratitis^16^. In the tectonic keratoplasty group, preoperative and final spherical equivalent (SE), and preoperative and final mean keratometry (K), were also recorded.

### Donor tissue preparation

Cornea donor tissue remnants were stored in Optisol-GS (Bausch & Lomb, Irvine, CA, USA) storage media at -80℃ deep freezer in-house eye bank immediately after initial donor tissue preparation. The donor cornea donated by the deceased donor was stored in 4℃ in Optisol-GS bottle before primary grafting and transplanted within 1 week. Depending on the surgical purpose, the donor cornea was processed for PKP or DSAEK during primary surgery, and the remaining corneoscleral button after PKP or anterior stromal lenticule after DSAEK was transferred back to the Optisol-GS bottle and cryopreserved in -80℃ deep freezer. Since we tried to remove the viable corneal cells through a freeze–thaw cycle, we did not use another medium for cryopreservation, but just frozen them in Optisol-GS. All procedures from enucleation of the donor eye to cryopreservation were performed aseptically in the operating room. To prevent cross contamination, the remnant tissue was separated in the operating room and was immediately cryopreserved, and the fresh tissue was grafted immediately. When a small graft was required in the peripheral region, or in the case of lesions involving corneoscleral tissue together, the corneoscleral rim of the PKP donor was cut and grafted for improved cosmetic results and histological uniformity. If a relatively large graft with a diameter of 5.0 mm or more and 8.25 mm or less was required or if deep anterior lamellar keratoplasty (DALK) was possible due to undamaged Descemet membrane in tectonic keratoplasty, the anterior stroma of the DSAEK donor was isolated and transplanted. Donor tissue remnants were thawed at room temperature 1 h before surgery. Except for the type of surgery applied to the donor cornea (PKP or DSAEK), information on the donor cornea such as donor information or freezing period was not recorded and could not be collected.

### Surgical procedures

**Figure 1:**
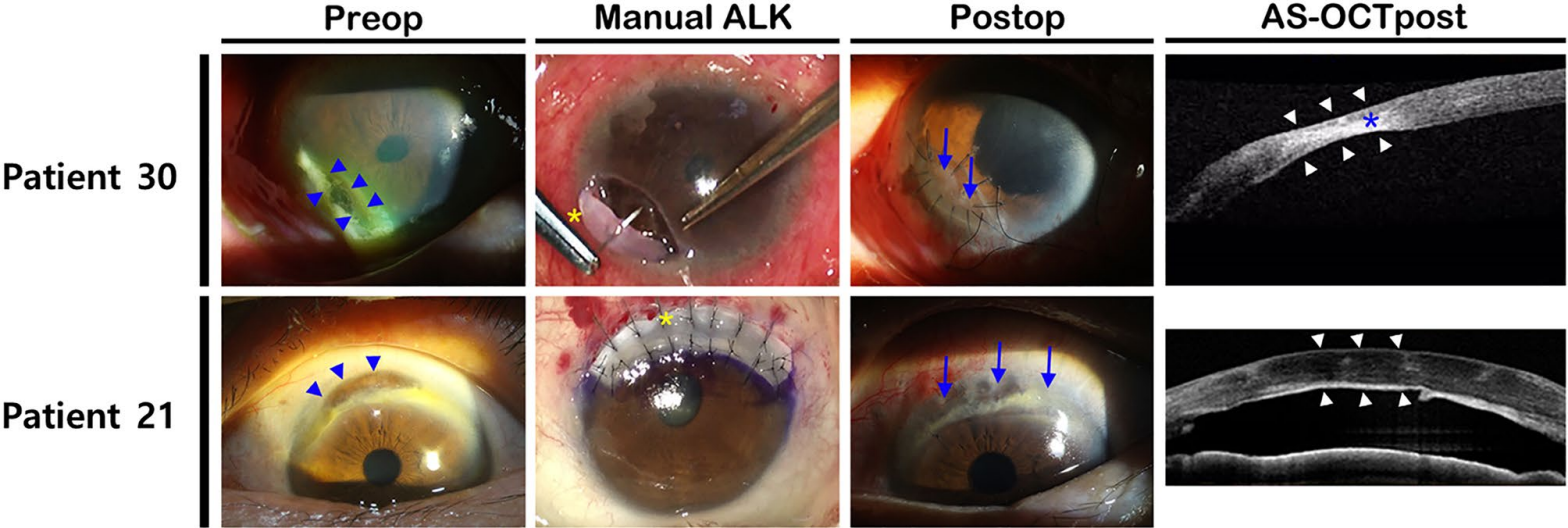
Cases of impending perforation at the peripheral cornea. (Upper) A case of marginal corneal melting with exposed Descemet membrane (blue arrowheads) due to chronic dacryocystitis. A manually trimmed graft was applied (blue arrows). Although the grafted cornea was slightly absorbed after tectonic keratoplasty due to sustained inflammation, further thinning did not occur after therapeutic dacrycystorhinostomy (white arrowheads). (Lower) A case of tectonic deep anterior lamellar keratoplasty due to Terrien marginal degeneration (blue arrowheads). Because extensive transplantation of the peripheral cornea was required, lamellar keratoplasty by fresh donors was contraindicated due to the risk of transplant rejection. The recipient stroma was removed up to the Descemet membrane level with manual dissection, and the corneal rim remaining after PKP was cut and transplanted (blue arrows). AS-OCT was performed 1 year postoperatively, revealing that corneal thickness was well maintained (white arrowheads). Preop—preoperative; ALK—anterior lamellar keratoplasty; Postop—postoperative; AS-OCTpost—postoperative anterior segment optical coherence tomography; PKP—penetrating keratoplasty; AS-OCT—anterior segment optical coherence tomography.

For small tectonic grafts, manually dissected anterior lamellar keratoplasty (ALK) was conducted. A penetrating graft was used for therapeutic keratoplasty, as the remaining deep infection source could induce recurrent and resistant infectious keratitis. Recipient beds were trephined using 3.0–6.0 mm skin biopsy punches (Stiefel Laboratories, Inc., Middlesex, United Kingdom) or using 7.0–8.0 mm diameter vacuum trephine and vacuum donor punch (Moria, Antony, France) along the size and location of underlying lesions. For DALK, the big bubble technique or viscodissection was used for recipient bed preparation^17^. For elongated lesions that could not be grafted with commercially available punches or trephine, such as Terrien marginal degeneration or marginal keratitis, recipient lesions were manually resected using a no. 15 bard parker or crescent blade until Descemet membrane depth in ALK or full thickness depth in PKP cases was reached (Fig. 1). Donor corneas were resected using the same diameter biopsy or donor punches in small grafts, but the donor graft was cut 0.25 mm larger than the recipient area in large diameter deep anterior lamellar grafts. Interrupted sutures using 10–0 nylon were placed in most cases and continuous sutures were placed in a few cases for fixation of graft tissue. Concurrent intracameral antibiotic injection was performed primarily for infectious corneal lesions, and temporary AMT was also performed to promote epithelial recovery during surgery for tectonic grafts with low risk of infection. Sutures were removed sequentially from 3 month after surgery.

### Postoperative medications

After grafting, topical levofloxacin antibiotic (Cravit, Santen Pharmaceutical Co., Ltd., Osaka, Japan) and steroid (Predforte, Allergan plc. Dublin, Ireland) eyedrops were used every 2 h in non-infectious lesions until complete epithelial coverage over the graft was attained. Immunosuppressive agents were not used due to the low risk of graft rejection by intended acellularity after the deep freeze–thaw procedure. After therapeutic grafting in microbial keratitis, preoperative empirical antimicrobial eyedrops were continued without topical steroids. Topical antimicrobial agents were changed to target the proper susceptible agents if indicated by postoperative microbial culture results.

### Statistical analysis

The descriptive statistics of the patients at the time of surgery were analyzed after data collected. Normality was tested for age, follow-up period, duration from diagnosis to surgery, graft size, preoperative and postoperative UCVA using the Shapiro–Wilk test. In all patients, preoperative and postoperative UCVA showed a normal distribution (*P* = 0.074 and 0.962, respectively). In the therapeutic keratoplasty group, age, graft size, preoperative and postoperative UCVA exhibited normal distributions (*P* = 0.920, 0.558, 0.087, and 0.063, respectively), while follow-up period and duration from diagnosis to surgery did not exhibit normal distributions (*P* < 0.001 and = 0.001, respectively). In the tectonic keratoplasty group, follow-up period, duration from diagnosis to surgery, graft size, and preoperative UCVA did not follow a normal distribution, (*P* < 0.001, 0.001, = 0.001, and = 0.032, respectively), but age and postoperative UCVA exhibited normal distributions (*P* = 0.420 and 0.061, respectively). No outliers were found for all variables. For statistical analysis to compare preoperative versus postoperative UCVA, paired Student’s t-test was used when the variable followed normality, and Wilcoxon signed-rank test was used when the variable did not follow normality. In comparisons between each subgroup, quantitative variables were compared using independent t-tests when the variable followed normality and Mann Whitney tests when the variable did not follow normality. Qualitative variables were compared using a chi-square test. P-values < 0.05 were considered statistically significant. Kaplan–Meier Survival Analysis was performed for each subgroup of enucleated patients. All statistical analyses were performed using IBM SPSS Statistics 25.0.0 (IBM Co., Armonk, NY, USA).

## Results

### Baseline characteristics

**Figure 2:**
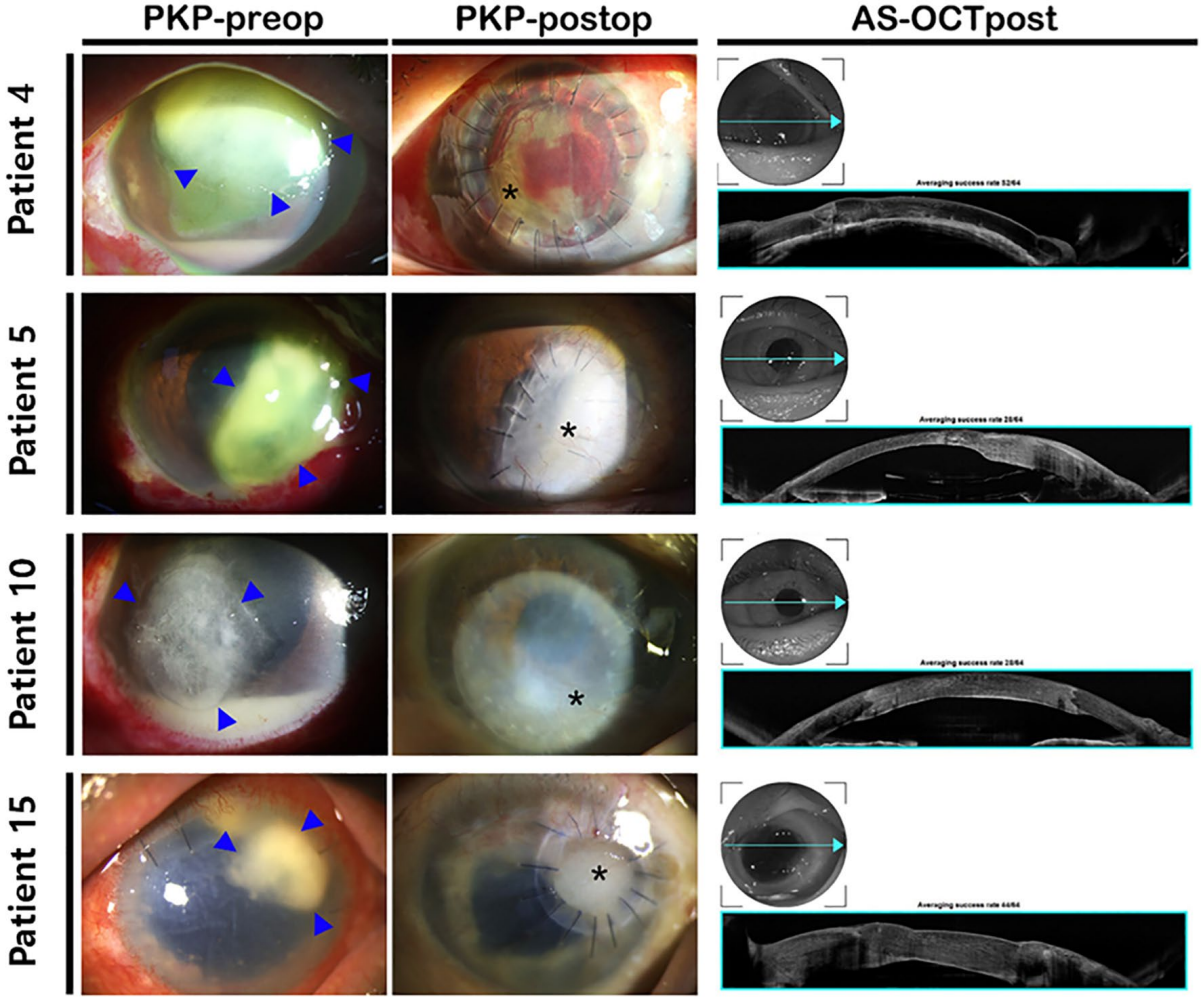
Representative images of refractory infectious keratitis and therapeutic PKP using cryopreserved cornea grafting. Although a large area of opaque cornea remained after PKP, the infection focuses were completely removed, and the anatomical integrity of the eye was successfully maintained. Optical keratoplasty was not performed because the patients did not desire further surgery. Postoperative AS-OCT revealed good restoration of corneal integrity. PKP—penetrating keratoplasty; Preop—preoperative; Postop—postoperative; AS-OCTpost—postoperative anterior segment optical coherence tomography; AS-OCT—anterior segment optical coherence tomography.

**Figure 3:**
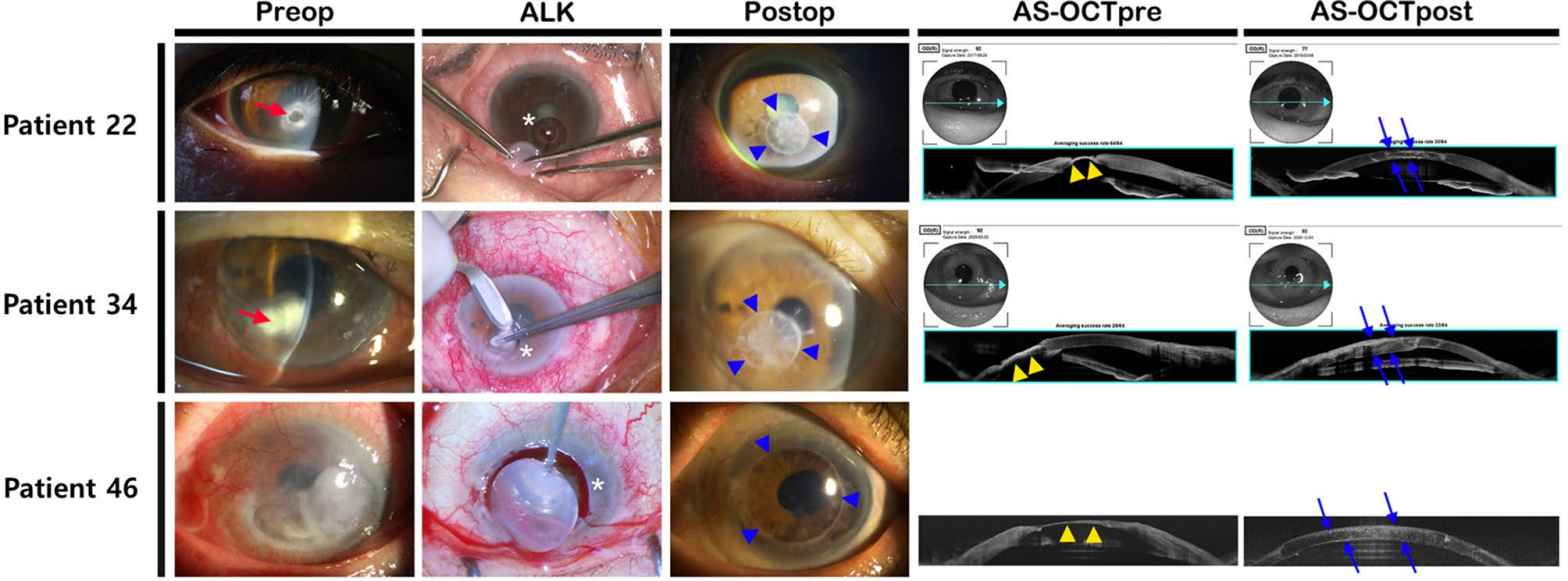
Representative cases of tectonic ALK using cryopreserved cornea. In the slit-lamp image, wide and deep ulceration was present (red arrows). During tectonic graft, careful dissection was performed as deeply as possible to remove the melted cornea (white asterisks). After surgery, the occurrence of corneal opacity was relatively low, yielding excellent cosmetic results (blue arrowheads). In preoperative AS-OCT, extremely thinned cornea was identified (yellow arrowheads). After keratoplasty, AS-OCT revealed good approximation of donor graft and recovery of corneal anatomy with curvature (blue arrows). Preop—preoperative; ALK—anterior lamellar keratoplasty; Postop—postoperative; AS-OCTpre—preoperative anterior segment optical coherence tomography; AS-OCTpost—postoperative anterior segment optical coherence tomography; AS-OCT—anterior segment optical coherence tomography.

Fifty-five cases from 46 patients were enrolled in the study. Mean patient age was 69.4 ± 14.6 years (range 33–95 years). There were 25 male and 21 female patients. Sixteen patients underwent surgery in the right eye, and thirty patients underwent surgery in the left eye. The average follow-up period was 23.7 ± 28.3 months (range 1–125 months). Twenty-three surgeries were performed in 18 patients for refractory infectious keratitis (Fig. 2) and thirty-two tectonic keratoplasty procedures were performed in 28 patients (Fig. 3). Among total cases, penetrating graft was performed in 17 cases, and ALK was performed in 38 cases. Corneoscleral remnants from PKP were used in 29 cases, and anterior lenticules from DSAEK preparation were used in 26 cases. The average duration between diagnosis and surgery was 7.4 ± 12.8 days (range 1–78 days), except for one patient (228 days), who received tectonic keratoplasty for cosmetic purposes. The average graft size was 5.34 ± 1.82 mm (range 3.00–8.25 mm) in total cases. The mean graft diameter was 3.70 ± 0.63 mm (range 3.00–5.00 mm) when PKP remnants were transplanted, and 6.87 ± 1.06 mm (range 4.90–8.25 mm) when DSAEK lenticules were transplanted. For patient number five with bacterial corneal ulcer, the longest (6.0 mm) and shortest (3.8 mm) axes were averaged to 4.90 mm.

With the exception of one patient in whom UCVA could not be measured due to Down’s syndrome, preoperative mean UCVA was 1.79 ± 0.92 LogMAR, and final UCVA was 1.53 ± 0.98 LogMAR. For low vision categories, CF was 1.9 (LogMAR), HM (hand motion at 30 cm) was 2.318–20, LP (light perception) was 2.7, and NLP (no light perception) was 3.018. Overall, final UCVA was significantly improved relative to preoperative UCVA in all patients (P = 0.005).

### Subgroup comparisons

In the comparison between each subgroup, no variable differed between groups, except that significantly more ALKs were performed in the tectonic keratoplasty group. The baseline characteristics and comparison analyses between subgroups are described in Table 1.

**Table 1:** Baseline patient characteristics Values are presented as mean ± standard deviation.

Baseline characteristics	Total	*P* value	Therapeutic keratoplasty		*P* value	Tectonic keratoplasty		*P* value	*P* value(Therapeutic vs. Tectonic)
No. of subjects/cases	46/55		18/23			28/32			
Age (years)	69.4 ± 14.6		70.9 ± 11.8 (50–88)			68.4 ± 16.3 (33–95)			0.565^c^
Gender (Male: Female)	25: 21		11: 7			14: 14			0.505^e^
Laterality (Right: Left)	16: 30		5: 13			11: 17			0.482^e^
Type of keratoplasty (PKP: ALK)	17: 38		15: 8			2: 30			** < 0.001**^**e**^
Donor leftover type (PKP: DSAEK)	29: 26		11: 12			18: 14			0.537^e^
Follow-up period (months)	23.7 ± 28.3		32.7 ± 37.4			19.1 ± 21.6			0.346^d^
Dx. to surgery duration (days)	7.4 ± 12.8		10.9 ± 17.0			4.9 ± 7.9			0.145^d^
Graft size (mm, PKP: DSAEK)	3.70 ± 0.63: 6.87 ± 1.06		3.67 ± 0.71: 6.85 ± 1.20			3.71 ± 0.61: 6.88 ± 0.96			0.591^d^
Mean UCVA (pre: postop)	1.79 ± 0.92: 1.53 ± 0.98	**0.005**^**a**^	1.82 ± 0.91: 1.85 ± 0.96		0.739	1.77 ± 0.94: 1.31 ± 0.95		**0.002**^**b**^	0.916^d^: 0.079^d^
Cause			Bacterial	7 (30.4%)		Impending perforation	21 (65.6%)		
			Fungal	14 (60.9%)		Apparent perforation	10 (31.3%)		
			Mixed	2 (8.7%)		Cosmetic	1 (3.1%)		

No.—number; PKP—penetrating keratoplasty; ALK—anterior lamellar keratoplasty; DSAEK—Descemet stripping automated endothelial keratoplasty; Dx.—diagnosis; UCVA—uncorrected visual acuity; LogMAR—logarithm of the minimal angle of resolution; pre—preoperative; postop—postoperative; N/A—not applicable.

^a^*P* value calculated using the.

^b^Paired Student’s t-test.

^c^Wilcoxon signed-rank test.

^d^Independent t-test.

^d^Mann Whitney test.

^e^Chi-square test.

Significant values are in [bold].

### Therapeutic keratoplasty

For patients who underwent therapeutic keratoplasty for refractory infectious keratitis, the causative organisms were bacterial in five cases (21.7%), fungal in 13 cases (56.5%), and mixed in two cases (8.7%). In three cases (13.1%), there was no detected microorganism in culture, or the test was not performed, but clinical signs were strongly suggestive of two bacterial (Patients 5 and 11) and one fungal keratitis case (Patient 18). The culture results of bacterial corneal ulcers identified infections from two *Staphylococcus* species, one *Micrococci* species, one *Klebsiella oxytoca*, one *Streptococcus pneumonia*, one *Enterococcus faecalis*, and one *Corynebacterium* species. For fungal keratitis, culture results identified infections from eight *Candida* species, one *Aspergillus flavus*, two *Fusarium* species, one *Alternaria* species, and one *Beauveria bassiana* infection. Patients 12 and 13 were diagnosed with mixed infectious corneal ulcer. *Pseudomonas aeruginosa* and filamentous fungi were isolated from Patient 12, and *Staphylococcus hominis* and *Candida albicans* were isolated from Patient 13.

Previously, six patients had undergone cataract surgery, one patient had undergone pterygium excision surgery, and two patients had undergone pars plana vitrectomy at least 6 months before keratoplasty. Four patients had undergone AMT, and two patients had previously received PKP using fresh cornea tissue, but this modality had failed. The mean diagnosis to surgery time was 10.9 ± 17.0 days (range 1–78 days), and the mean follow-up period was 32.7 ± 37.4 months (range 3–125 months).

### Surgical outcomes

**Figure 4:**
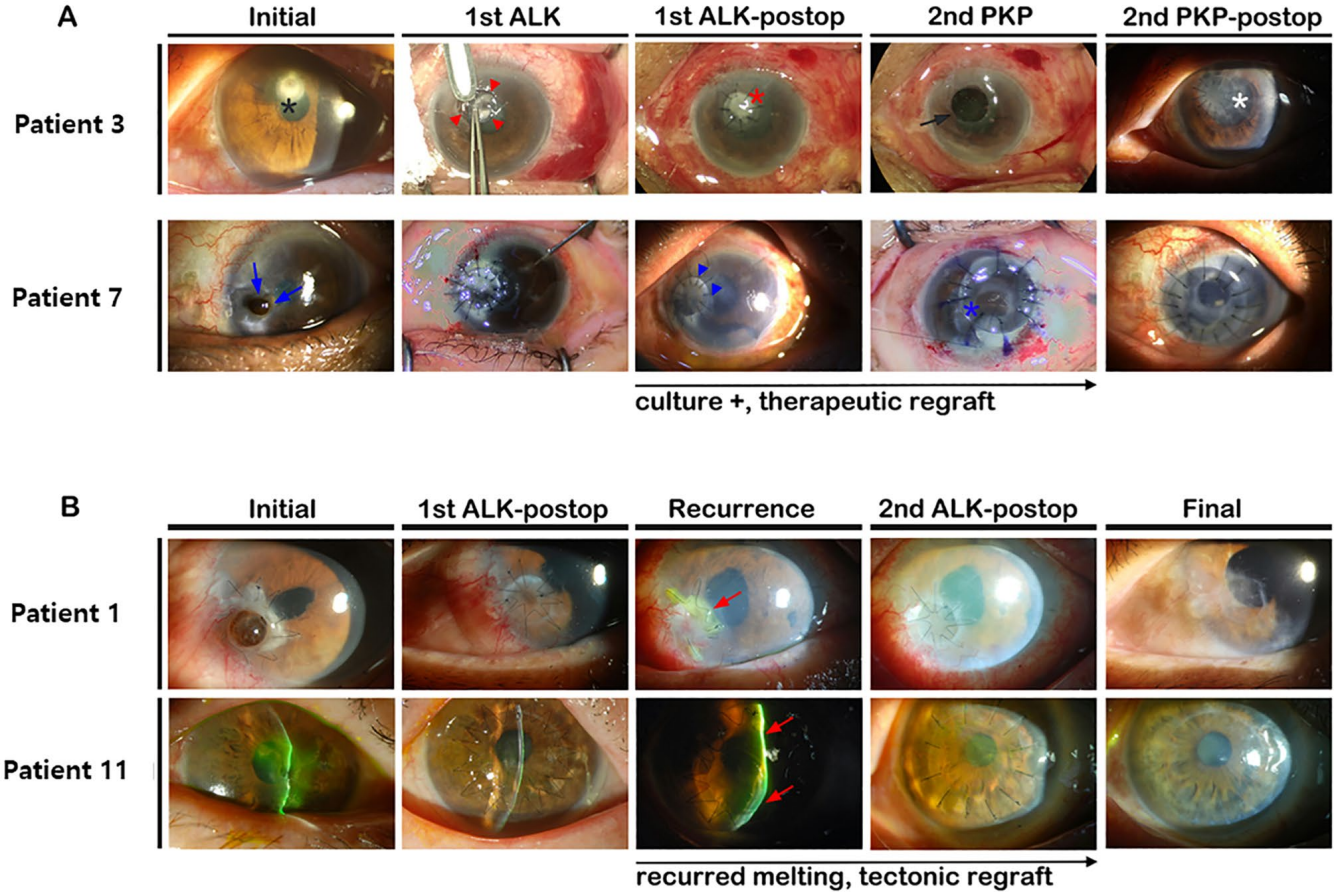
Representative images of patients who underwent repeated therapeutic or tectonic keratoplasty.** (A)** Patient 3 was a 77-year-old female with hepatocellular carcinoma and chronic obstructive pulmonary disease. Patient 7 was an 83-year-old female with uncontrolled diabetes and asthma. Both elderly patients were using steroid inhalers and had weakened immunity, so aggressive treatments were used throughout management of the case. A prompt therapeutic ALK was performed, but candidiasis recurred at the graft–host junction. To eradicate fungi, additional wide and full thickness therapeutic PKP was performed. **(B)** Patient 1 and 11. Primary tectonic keratoplasty was performed due to impending corneal perforation. Due to recurrence of corneal melting related to underlying systemic disease, a second lamellar graft was performed. ALK—anterior lamellar keratoplasty; Postop—postoperative; PKP—penetrating keratoplasty.

**Figure 5:**
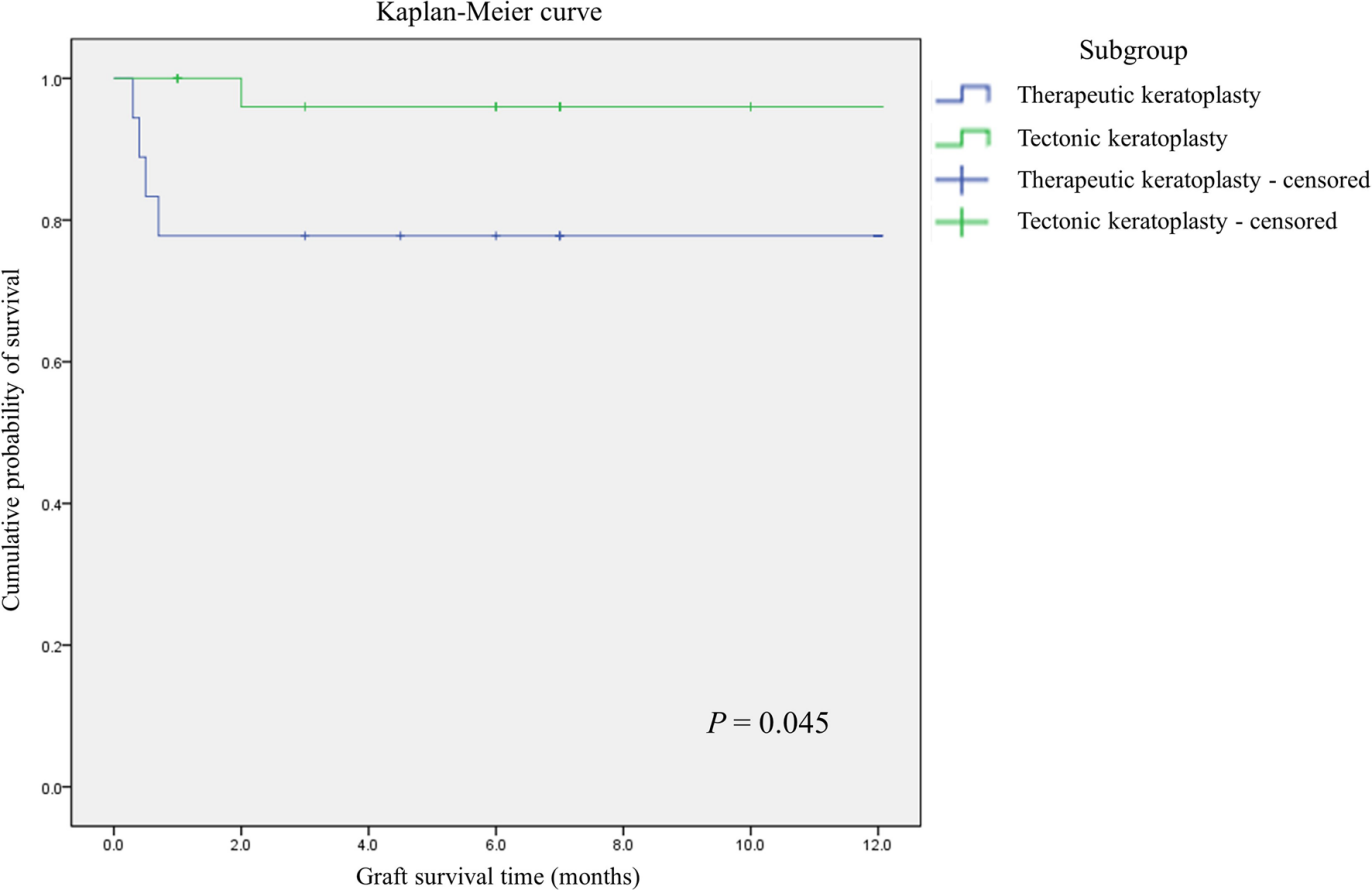
Kaplan–Meier curve for enucleated patients. When surgical success was defined as maintaining ocular integrity for more than 12 months, all patients that underwent enucleation were enucleated within 2 months after surgery, and all enucleations were performed within 1 month in the therapuetic keratoplasty group. In the group treated with therapeutic keratoplasty, the period of graft survival was significantly shorter, which eventually led to enucleation or eviceration. *P* value calculated using the Logrank test.

Four patients underwent an additional graft by PKP after ALK, and one patient underwent repeat DALK using cryopreserved cornea due to recurrence of microbial keratitis at the graft and host junction (Fig. 4). In the case of reoperation, the identified microorganism was confirmed as the same species before reoperation, so it is strongly suspected that it is a recurrence of an existing infection rather than caused by donor cornea. Four patients ultimately underwent enucleation or evisceration, primarily due to uncontrolled infection (Patients 2, 6, 8, and 9) (Fig. 5). Several patients exhibited longstanding epithelial defects related to vigorous topical antimicrobial treatment, but ocular integrity was ultimately maintained without additional surgical interventions. Mean pre- and postoperative UCVAs were 1.82 ± 0.91 LogMAR and 1.85 ± 0.96 LogMAR, respectively, which was not statistically significant (*P* = 0.739). These results are summarized in Tables 2 and 3.

**Table 2:** Preoperative demographics of patients undergoing therapeutic keratoplasty with cryopreserved cornea.

Patient No	Sex/Age	Laterality	Cultured organism(s)	Previous operation(s)	Interval (Dx. to surgery, days)	Keratoplasty procedure(s)
1	M/77	OS	*Candida albicans*	Cat, AMT	78	ALK
			*Candida albicans (recurred)*	Cat, AMT, ALK	7	PKP (repeated)
2	M/50	OS	*Micrococci species*	Cat	6	PKP
3	F/77	OS	*Candida albicans*	–	1	ALK
			*Candida albicans (recurred)*	ALK	4	PKP (repeated)
4	F/80	OS	*Streptococcus pneumoniae, enterococcus faecalis*	AMT	9	PKP
5	F/77	OD	Failed to identify	–	3	PKP
6	M/85	OS	*Staphylococcus aureus*	Cat, PKP	1	PKP
7	F/83	OS	*Candida parapsilosis*	Cat, Pterygium	3	ALK
			*Candida parapsilosis (recurred)*	Cat, Pterygium, ALK	1	PKP (repeated)
8	M/50	OS	*Staphylococcus aureus, Corynebacterium species*	Removal of corneal FB	5	PKP
9	M/56	OS	*Aspergillus flavus*	PKP	1	PKP
10	F/74	OD	*Fusarium species*	–	19	PKP
11	F/68	OD	N/A	–	2	ALK
			*Klebsiella Oxytoca*	ALK, AMT	31	PKP (repeated)
12	M/54	OD	*Pseudomonas aeruginosa,* Filamentous fungi	Corneal suture, AMT	5	PKP
13	M/73	OD	*Staphylococcus hominis, Candida albicans*	–	1	ALK
14	M/70	OS	*Alternaria species*	–	12	ALK
15	F/78	OS	*Fusarium oxysporum*	Cat, PPV, Removal of IOL	30	PKP
16	M/75	OS	*Beauveria bassiana*	Cat, PPV	4	PKP
17	M/62	OS	*Candida pelliculosa*	–	14	ALK
			*Candida pelliculosa (recurred)*	ALK	2	DALK (repeated)
18	M/88	OS	Failed to identify	–	11	PKP

No.—number; Dx—diagnosis; M—male; F—female; OD—oculus dexter; OS—oculus sinister; Cat—cataract surgery; AMT—amniotic membrane transplantation; PPV—pars plana vitrectomy; IOL—intraocular lens; ALK—anterior lamellar keratoplasty; PKP—penetrating keratoplasty; DALK—deep anterior lamellar keratoplasty; N/A—not applicable; FB—foreign body.

**Table 3:** Outcomes after therapeutic keratoplasty using cryopreserved cornea.

Patient No	UCVA (preop/final)	Type of donor	Graft size (mm)	Underlying dz. (s)	Follow-up duration (months)	Final graft status	Prognosis
1	0.8/0.15	PKP	3.00	Prostate cancer, cerebral infarction	45	Recurrence	Regraft
		PKP	3.00			Stable	Fair
2	NLP/NLP	PKP	3.00	Uncontrolled DM, chronic alcoholism, epilepsy	N/A	Graft melting	Enucleation
3	CF/CF	PKP	4.00	HCC, left pontine hemorrhage, COPD	7	Recurrence	Regraft
		PKP	4.00			Stable	Fair
4	NLP/NLP	DSAEK	7.75	Carotid stenosis, DM, cerebral infarction	19	Stable	Fair
5	1.2/1.0	DSAEK	6.0 × 3.8	DM	7	Stable	Fair
6	NLP/NLP	DSAEK	8.00	Cerebral infarction	N/A	Graft melting	Evisceration
7	1.1/1.2	PKP	4.00	Uncontrolled DM, asthma	4.5	Recurrence	Regraft
		DSAEK	7.25			Stable	Fair
8	NLP/NLP	PKP	4.00	Endophthalmitis	N/A	Graft melting	Enucleation
9	HM/LP	DSAEK	8.25	–	N/A	Graft melting	Enucleation
10	1.2/1.7	DSAEK	8.00	Active TB, asthma	48	Stable	Fair
11	1.0/1.2	DSAEK	6.50	RA with secondary SJS	125	Recurrence	Regraft
		DSAEK	6.50			Stable	Fair
12	HM/NLP	PKP	Manual	Uncontrolled DM, endophthalmitis	96	Stable	Fair
13	1.1/1.1	PKP	Manual	active TB	21	Stable	Fair
14	1.0/1.5	PKP	5.00	–	18	Stable	Fair
15	LP/CF	DSAEK	6.00	Arrhythmia, both TKR, hypothyroidism, endophthalmitis	7	Stable	Fair
16	HM/CF	DSAEK	6.00	DM	3	Stable	Fair
17	0.1/0.1	PKP	3.00	DM	51	Recurrence	Regraft
		DSAEK	8.00			Stable	Fair
18	1.7/CF	DSAEK	5.00	Stomach cancer, prostate cancer, RUL BCC	6	Stable	Fair

No.—number; UCVA—uncorrected visual acuity; preop—preoperative; dz.—disease; F/U—follow-up; NLP—no light perception; LP—light perception; HM—hand motion at 30 cm; CF—counting fingers at 30 cm; PKP—penetrating keratoplasty; DSAEK—Descemet stripping automated endothelial keratoplasty; DM—diabetes mellitus; HCC, hepatocellular carcinoma; COPD, chronic obstruction pulmonary disease; TB, tuberculosis; RA, rheumatoid arthritis; SJS, Stevens–Johnson syndrome; TKR—total knee replacement; RUL BCC—right upper eyelid basal cell carcinoma; N/A—not applicable.

*UCVA presented as logarithm of the minimal angle of resolution.

### Tectonic keratoplasty

Preoperative conditions for tectonic keratoplasty included 21 impending corneal perforations (65.6%), ten apparent corneal perforations (31.3%), and one thick central corneal opacity (3.1%) (Table 4).

**Table 4:** Preoperative demographics of patients undergoing tectonic keratoplasty using cryopreserved cornea.

Patient No	Sex/Age	Laterality	Pre-existing disease	Previous operation(s)	Interval (Dx. to surgery, days)	Keratoplasty procedure
19	F/52	OS	Marginal keratitis impending perforation	AMT	7	ALK
20	M/64	OS	Corneoscleral impending perforation	–	7	ALK
21	F/62	OS	Terrien marginal degeneration	–	7	ALK
22	F/52	OD	Impending corneal perforation	–	1	ALK
23	F/64	OD	Corneal perforation	–	44	ALK
24	M/77	OD	Corneal perforation	AMT	1	ALK
25	M/63	OD	Corneoscleral perforation	–	3	ALK
26	F/95	OS	Necrotizing sclerokeratitis	Cat, Pterygium	10	ALK
27	M/65	OD	Herpes keratitis impending perforation	–	2	ALK
			Graft melting	ALK	7	ALK (repeated)
28	M/71	OS	Neurotropic ulcer impending perforation	Repair of orbital wall fracture	2	ALK
29	F/78	OS	Herpes keratitis corneal perforation	AMT	4	ALK
30	M/86	OD	Descemetocele	–	2	ALK
31	F/82	OS	Impending corneal perforation	Cat, AMT	1	ALK
			Graft melting	ALK	1	PKP (repeated)
32	M/87	OS	Descemetocele	ICCE c PCLI	1	ALK
33	M/48	OD	Corneal perforation	PPV, IOFB, S-oil	2	ALK
			Graft melting	ALK	4	ALK (repeated)
			Graft melting	ALK	4	PKP (repeated)
34	M/75	OD	Impending corneal perforation	–	3	ALK
35	F/90	OS	Descemetocele	–	14	ALK
36	M/33	OS	Traumatic corneoscleral perforation	Scleral suture, Iridoplasty	2	ALK
37	M/58	OS	Central corneal opacity	PPV, S-oil	228	DALK
38	F/41	OS	Cornea perforation	PKP, Cat, synechiolysis	1	ALK
39	M/68	OS	Impending corneal perforation	–	1	ALK
40	F/90	OD	Impending corneal perforation	Cat	4	ALK
41	F/80	OS	Impending corneal perforation	Cat	1	ALK
42	M/68	OS	Corneal perforation	–	3	ALK
43	F/39	OD	Exposure keratitis corneal perforation	–	3	ALK
44	F/78	OS	Herpes keratitis corneal perforation	AMT	5	ALK
45	M/65	OD	Herpes keratitis impending perforation	–	2	ALK
46	F/83	OS	Impending corneal perforation	Cat	2	ALK

No.—number; Dx.—diagnosis; M—male; F, female; OD—oculus dexter; OS—oculus sinister; Cat—cataract surgery; AMT—amniotic membrane transplantation; ICCE c PCL—intracapsular cataract extraction with posterior capsular lens implantation; PPV—pars plana vitrectomy; IOFB—intraocular foreign body; S-oil—silicone oil; ALK—anterior lamellar keratoplasty; PKP—penetrating keratoplasty; DALK, deep anterior lamellar keratoplasty.

**Figure 6:**
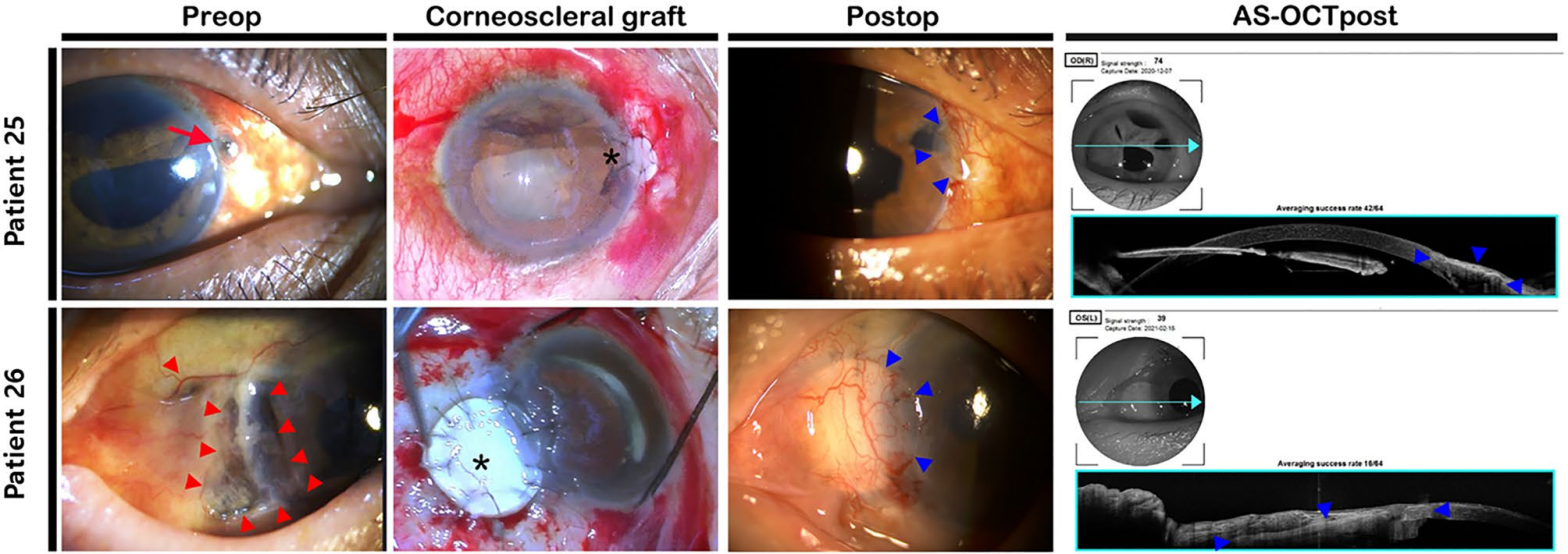
Representative cases of corneoscleral graft. Patient 25 presented with a relatively large diameter corneoscleral penetration site at the 3 o’clock position (red arrow). A corneoscleral graft using a remnant rim of corneal donor remnant tissue was well fitted to the penetration site (asterisk, top row). Postoperative slit-lamp examination and AS-OCT revealed good cosmesis with well-formed corneoscleral integrity (blue arrowheads). Patient 26 presented with widespread corneoscleral melting related to necrotizing sclerokeratitis (red arrowheads). Partial thickness corneoscleral graft and conjunctival rotation autograft were performed (asterisk). The grafts aligned to the corneal and scleral borders of the limbus, and had good cosmetic and anatomical outcomes (blue arrowheads). Preop—preoperative; Postop—postoperative; AS-OCTpost—postoperative anterior segment optical coherence tomography; AS-OCT—anterior segment optical coherence tomography.

**Figure 7:**
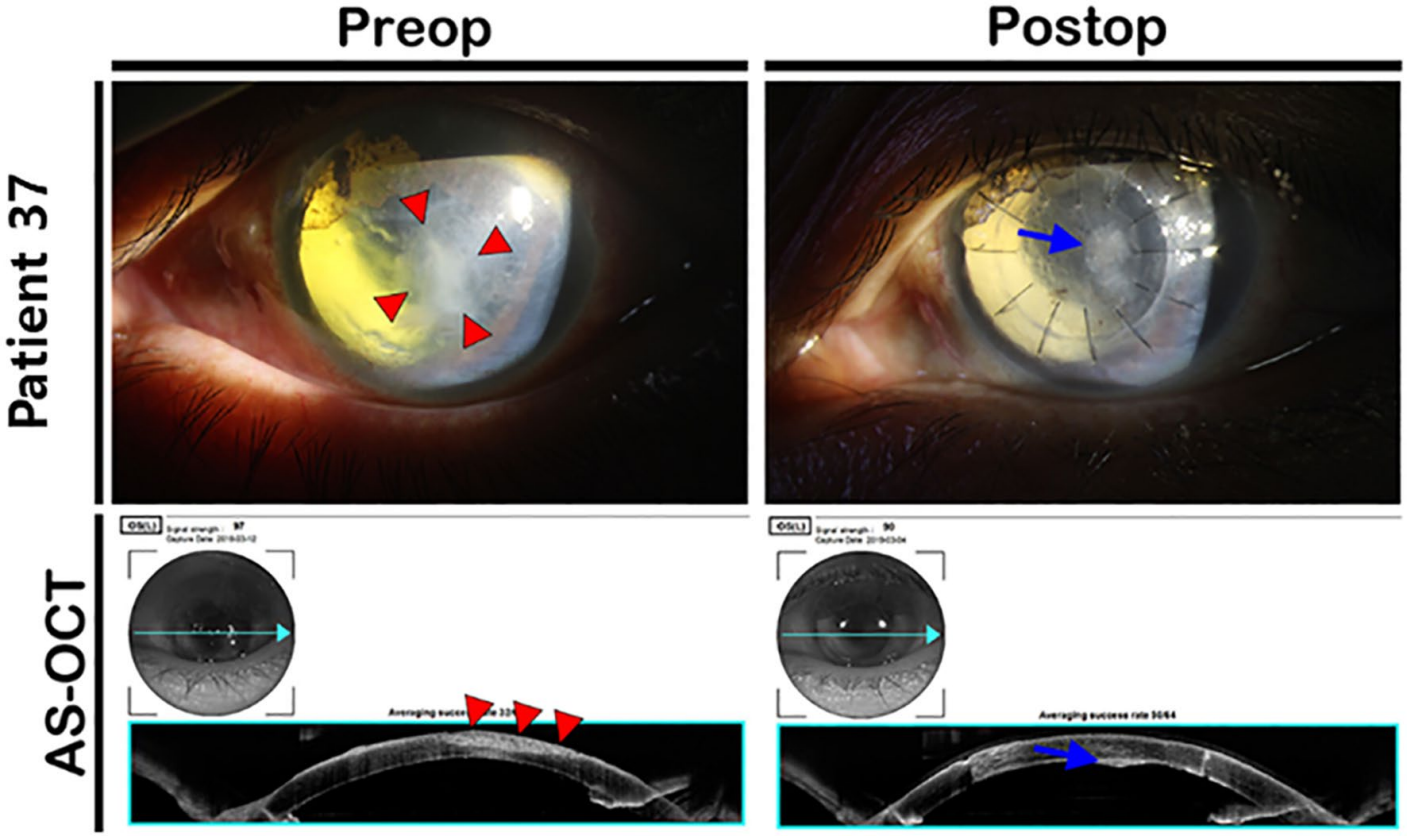
Cosmetic DALK for thick central corneal opacity. Preoperatively, a distinctive thick central corneal opacity was present (red arrowheads). After DALK using cryopreserved cornea, corneal opacity was significantly decreased. A thin opacity was observed near the Descemet membrane, but corneal clarity was significantly improved. Preop—preoperative; Postop—postoperative; AS-OCT—anterior segment optical coherence tomography; DALK—deep anterior lamellar keratoplasty.

Because lamellar graft patching is known as a safe and effective approach to treatment for non-infectious corneal perforations^21^, all patients received an anterior lamellar graft at initial surgery. However, Patients 31 and 33 received PKP for the second and third grafts (Table 4). Patient 37 underwent cosmetic ALK due to a centrally positioned thick corneal opacity. Corneoscleral grafts were performed to maintain histological identity and cosmetic suitability in patients with corneoscleral melting of necrotizing sclerokeratitis or widespread lacerations with tissue defect (Patients 25, 26, and 36) (Fig. 6). Patient 37 received cosmetic DALK for a thick central corneal opacity after repeated pars plana vitrectomy due to retinal detachment. After cosmetic DALK using cryopreserved corneal tissue, the opacity over the stroma was almost eliminated, and the transparency of the transplanted cornea was improved. The patient was satisfied with the appearance (Fig. 7).

Five patients had undergone permanent and/or temporary AMT prior to keratoplasty, but AMT had failed due to graft melting. The mean time from diagnosis to surgery was 4.9 ± 7.9 days (range 1–44 days, with the exception of Patient 37 for whom the mean time to surgery was 228 days), and the mean follow-up period was 19.1 ± 21.6 months (range 1–81 months).

### Surgical outcomes

Three patients underwent repeated tectonic grafts due to progressive graft thinning and perforation (Patients 27, 31, and 33). Patient 33 underwent two repeated grafts due to uncontrolled graft melting, but ocular integrity was ultimately established after the third graft. Patient 38 refused regraft and elected to undergo enucleation due to graft thinning (Fig. 5). Finally, of the 28 patients who underwent tectonic graft, ocular integrity was successfully maintained in 27 patients (Table 5). Mean preoperative UCVA was 1.77 ± 0.94 LogMAR, and postoperative UCVA was 1.31 ± 0.95 LogMAR. After tectonic graft, UCVA improved significantly, although this technique is a single-step surgical procedure without optical keratoplasty (*P* = 0.001). Preoperative SE was -1.32 ± 1.62 diopter (D), and postoperative SE was -1.26 ± 1.33 D. Preoperative mean K was 44.27 ± 1.72 D, and postoperative mean K was 44.94 ± 2.44 D. These results were not statistically significantly (*P* = 0.905 and 0.937, respectively). These results are summarized in Tables 4 and 5.

**Table 5:** Outcomes after tectonic keratoplasty using cryopreserved cornea.

Patient No	UCVA (Preop/Final)	SE (Preop/Final, D)	Mean K (Preop/Final, D)	Corneal astigmatism (Preop/Final, D)	Type of donor	Graft size (mm)	Underlying dz. (s)	F/U duration (months)	Final graft status	Prognosis
19	0.4/0.0	− 3.50/− 1.88	N/A	UCK	PKP	3.00	Uncontrolled DM	23	Stable	Fair
20	1.0/0.5	− 4.00/− 0.13	43.63/43.62	1.50/1.00	PKP	4.00	–	7	Stable	Fair
21	0.0/0.0	− 0.25/− 0.75	N/A/47.13	–/3.75	PKP	Manual	–	62	Stable	Fair
22	1.2/0.3	− 0.63/− 3.63	fail/45.63	–/5.25	PKP	4.00	–	19	Stable	Fair
23	1.2/0.1	fail/0.38	44.88/45.75	1.25/1.00	PKP	4.00	–	36	Stable	Fair
24	1.1/0.4	− 2.25/− 1.88	45.38/46.50	4.25/4.50	PKP	4.00	COPD, pancytopenia	15	Stable	Fair
25	0.3/0.1	− 0.13/0.75	46.13/43.63	2.75/1.25	PKP	3.00	–	10	Stable	Fair
26	0.3/0.8	− 0.88/− 1.75	45.75/46.00	2.50/3.50	DSAEK	6.00	Elderly	10	Stable	Fair
27	HM/1.7	fail/fail	42.50/fail	1.00/–	DSAEK	7.50	–	57	Graft melting	Regraft
					DSAEK	Unknown			Stable	Fair
28	HM/1.1	fail/fail	N/A	UCK	PKP	4.00	Elderly	1	Stable	F/U loss
29	1.4/1.7	fail/fail	N/A	UCK	PKP	4.00	RA	1	Stable	F/U loss
30	CF/1.2	fail/− 2.88	N/A	UCK	PKP	Manual	Uncontrolled DM	3	Graft melting	Fair
31	NLP/NLP	fail/fail	N/A/fail	UCK	DSAEK	7.25	MI, liver abscess, SAH	81	Graft melting	Regraft
					DSAEK	6.00			Stable	Fair
32	HM/CF	− 1.13/fail	N/A/39.63	− /5.50	PKP	5.00	CHF, tSDH, thrombocytopenia	7	Recurrent erosion	Fair
33	LP/HM	fail/fail	N/A/fail	UCK	PKP	4.00	IOFB, RD	33	Graft melting	Regraft
					DSAEK	5.00			Graft melting	Regraft
					DSAEK	6.00			Stable	Fair
34	1.1/CF	fail/fail	41.63/fail	3.25/–	PKP	3.00	Gout	7	Stable	Fair
35	LP/HM	fail/fail	fail/fail	UCK	DSAEK	8.00	Elderly	6	Stable	Fair
36	CF/0.05	fail/fail	fail/fail	UCK	PKP	3.00	–	12	Stable	Fair
37	NLP/NLP	fail/fail	fail/48.13	–/4.75	DSAEK	6.00	IOFB, RD	28	Stable	Fair
38	UCK/UCK	UCK/UCK	UCK/UCK	UCK	PKP	Unknown	Down’s syndrome	N/A	Graft thinning	Enucleation
39	0.5/1.0	0.88/− 0.75	N/A	UCK	PKP	Manual	Leprosy, MI	7	Stable	Fair
40	NLP/1.7	fail/fail	fail/fail	UCK	DSAEK	8.00	ILD, stroke, elderly	7	Stable	Fair
41	LP/HM	fail/fail	fail/fail	UCK	DSAEK	7.25	DM, TKR, spine fx	7	Stable	Fair
42	HM/HM	fail/fail	fail/fail	UCK	DSAEK	7.50	–	7	Stable	Fair
43	CF/0.9	fail/fail	fail/fail	UCK	PKP	4.00	SAH with hemiparesis	6	Stable	Fair
44	LP/CF	fail/fail	fail/fail	UCK	DSAEK	7.75	uncontrolled DM	1	Stable	Fair
45	HM/HM	UCK/UCK	fail/fail	UCK	PKP	3.00	–	57	Stable	Fair
46	HM/0.7	fail/− 1.38	fail/43.38	–/1.25	DSAEK	7.25	Arrhythmia	6	Stable	Fair

No.—number; UCVA—uncorrected visual acuity; Preop—preoperative; SE—spherical equivalent; D—diopter; K—keratometry; dz.—disease; F/U—follow-up; NLP—no light perception; LP—light perception; HM—hand motion at 30 cm; CF—counting fingers at 30 cm; UCK—uncheckable; N/A—not applicable; PKP—penetrating keratoplasty; DSAEK—Descemet stripping automated endothelial keratoplasty; DM—diabetes mellitus; COPD—chronic obstruction pulmonary disease; RA—rheumatoid arthritis; MI—myocardial infarction; CHF—congestive heart failure; tSDH—traumatic subdural hemorrhage; IOFB—intraocular foreign body; RD—retinal detachment; ILD—interstitial lung disease; TKR—total knee replacement; fx.—fracture; SAH—subarachnoid hemorrhage.

*UCVA presented as the logarithm of the minimal angle of resolution.

## Discussion

The present case series aimed to determine if therapeutic or tectonic corneal grafts using cryopreserved donor tissue remnants are viable treatment options for refractory infectious keratitis or apparent/impending corneal perforation. Most of the patients included in the study required emergency surgery, and many patients were elderly. Therefore, in order to remove the infection and, if possible, achieve the desired outcome of improved vision, we opted to perform corneal transplantation using cryopreserved corneas due to their availability for emergent procedures. Until recently, transplantation of fresh cornea tissue has been considered most appropriate in these cases if the donor cornea supply is sufficient. Sharma S. et al. reported that both functional and anatomical success were achieved for an average of 11 months in about 60% of patients with corneal ulceration, thinning, and perforation after corneal patch graft^22^. However, cornea donor tissue is severely lacking in Asian countries^23–25^. From a non-medical point of view, Asian countries suffer from a significant shortage of organ donors due to deep-rooted ethnical and religious beliefs. This obstacle will be difficult to overcome for the foreseeable future^5^. Cornea tissues are now available from eye banks in countries with more abundant corneal donors, such as the United States. However, even in this case, there are significant limitations due to the high cost and limited availability of fresh tissue. In addition, the shortage of fresh corneal tissues worldwide is getting serious at a time of global crisis when the world is facing COVID-19^26^. Therefore, this strategy is insufficient to address the current shortage of donor tissue.

On the other hand, it is questionable whether fresh corneal tissue is the most appropriate for all transplants when the availability of fresh tissue is severely lacking. Therapeutic grafts for infectious lesions or tectonic grafts using fresh cornea for lesions existing in the corneal periphery pose additional problems such as reinfection or graft rejection. In addition, the use of immunosuppressants after transplantation, as would be necessary with the use of fresh tissue, can lead to uncontrollable systemic side effects and exacerbate existing physiological problems in elderly patients with poor medical condition, which is the patient cohort that most commonly requires tectonic or therapeutic keratoplasty for perforated corneas or refractory infectious keratitis. In these contexts, prompt intervention for infectious corneal ulcer and impending corneal perforation preclude the use of fresh donor tissue, which is not always available. Therefore, prior studies have evaluated the feasibility of conducting tectonic or therapeutic PKP or lamellar keratectomy (LK) using glycerol-preserved donor corneas^13,27,28^. Storage of corneal tissue in glycerol has advantages such as storage at room temperature and low probability of graft rejection due to acellularity caused by glycerol. However, when glycerol-preserved cornea is used in cases of infectious keratitis, reinfection may occur depending on the causative microorganism or secondary glaucoma may occur^29^.

More recently, tectonic keratoplasty using femtosecond laser intrastromal lenticules^30^ and therapeutic xenografts^31^ have been proposed as new therapeutic modalities. Although lenticule size could be sufficient to create tectonic grafts for keratoplasty, this modality is difficult to apply in cases with deep or large ulcerations due to limitations in the size and thickness of the lenticules^32^. In the case of xenografts, the probability of immune rejection is high, and a high degree of immunosuppression is therefore required. This modality would thus be contraindicated for the targeted patient population, in which poor medical condition is common, and immunosuppression would pose significant risk of reinfection in infectious keratitis^33,34^.

The long-term preservation method of donor tissue remnants is a major consideration in the use of preserved corneal tissue for keratoplasty. In previous reports, the advantages of glycerol storage have been reported. Glycerol storage at room temperature lowers the antigenicity of donor tissue by destroying endothelial cells through osmotic stress, and the tissue can be inexpensively stored over long periods of time^9,35^. However, as glycerol is a dehydrating agent with antimicrobial and antiprotease properties, it can damage corneal endothelial cells in the recipient eyes. When storing tissues remnants in glycerol, the storage media must be replaced with water before use, and caution is required because glycerol remains in the tissue and spreads to the ocular surface or anterior chamber, where it can cause serious damage to the intraocular tissues^36,37^. In addition, there are no data to support the safety of glycerol application to ocular tissues. On the other hand, Optisol-GS preserves the corneal epithelium at 4 °C for more than 2 weeks, and has an antibiotic effect due to the use of gentamicin (100 μg/mL) and streptomycin (200 μg/mL) in this formula. Optisol-GS has been widely used for tissue storage and transplantation, and there are sufficient data to support its safety^38,39^. Since Optisol-GS is not a cryoprotectant, osmotic stress of cells and tissue freezing injury may occur if the remnant cornea is immersed in Optosol-GS for rapid freezing^35^. However in this process, the endothelial cells are definitely destroyed, so we thought it would be more helpful to prevent immune rejection. Therefore, we selected Optisol-GS as the preservative medium for cryopreservation of donor tissue remnants. Our results suggest that this approach has several benefits over previously used methods.

In this study, the mean age of patients who underwent graft was 69.4 years, and 26.1% of patients were over 80 years old. Therefore, the patients were elderly, and most had underlying medical conditions. Within the patient cohort, 11 patients (24.4%) had uncontrolled diabetes, and five (11.1%) had a history of stroke. One patient (2.2%) had hemiplegia, and one patient (2.2%) had intellectual disability. In general, high-dose steroids are needed to prevent graft rejection in corneal transplant^40^. However, in older patients, the use of immunosuppressants could aggravate cardiovascular risk, and cause malignancy and infections due to over-immunosuppression in long-term therapy^41,42^. Immunosuppressants generally used for solid organ transplantations, such as cyclosporine, tacrolimus, and glucocorticoids, could exacerbate high blood pressure or blood sugar in this vulnerable patient population^43,44^. In addition, the use of immunosuppressive drugs in patients with a history of stroke poses a significant infection risk^45,46^. Therefore, immunosuppressants should be used with significant caution in the elderly, patients with underlying diseases such as diabetes and hypertension, and patients who have suffered a stroke. In our case series, only topical antibiotics and steroid eye drops were used, and no systemic treatments were administered. Nevertheless, no graft rejection occurred in either tectonic or therapeutic grafts, suggesting that sufficient acellularity can be obtained only by deep freezing cornea donor tissue in Optisol-GS. Finally, anatomical success was achieved in 41 of 46 patients (89.1%). Anatomical integrity was maintained in 14 of 18 patients (77.8%) in the therapeutic keratoplasty group, and 27 of 28 patients (96.4%) in the tectonic keratoplasty group. Most of the therapeutic keratoplasty cases were elderly patients in poor general condition, with co-existing conditions such as cancers or uncontrolled diabetes. In the present study, because therapeutic keratoplasty was performed for refractory keratitis in which most of the patients did not respond to drugs for fungal keratitis, the success rate of approximately 80% is remarkable. Although reinfection occurred after the initial graft in five patients, the infection was controlled, and corneal integrity was successfully maintained after the patients underwent an additional therapeutic graft.

Particularly in corneal lesions located near the limbus, such as Mooren’s ulcer, Terrien’s marginal degeneration, or staphylococcal marginal keratitis, the incidence of graft rejection is high, ranging from 39.4 to 64% after keratolimbal allograft transplantation^47,48^. In the present study, nine patients with keratitis invading the marginal cornea or corneoscleral tissue were identified. All of the patients maintained successful anatomical integrity after grafting using cryopreserved cornea without rejection. In general, eccentric grafts have a higher risk of rejection than central position grafts because they are proximal to the vascularized limbus and are thus surrounded by host corneal tissue rich in Langerhans cells^49^. Therefore, for marginal lesions, it is more effective to use cryopreserved cornea than fresh cornea to reduce antigenicity.

In cases of infectious keratitis, the use of topical or systemic immunosuppressants after grafting could promote recurrence or exacerbation of the infection^7,8^. In addition, systemic broad-spectrum antimicrobial agents must be used for at least 2 weeks postoperatively until the corneal epithelium has healed^50^. If systemic immunosuppressants and antibiotics are used together, patients are at risk for severe nephrotoxicity and hepatotoxicity, especially in elderly cohorts^51,52^. Because the prevalence of infectious keratitis is relatively high in elderly and medically compromised patients, it would be inappropriate to use fresh cornea for therapeutic keratoplasty in these cases^53,54^. In fact, in the present study, the average age of patients who underwent therapeutic keratoplasty for infectious corneal ulcers was 70.9 years, and major medical conditions were present in most patients. Considering these factors, acellularized grafting of cryopreserved cornea is preferable for therapeutic keratoplasty, as the risk of systemic side effects due to immunosuppressant use is decreased.

General anesthesia must be used for elderly corneal transplantation patients with dementia or poor cooperation, and for patients with intellectual disabilities. The probability of postoperative neurological and cardiac complications is increased when elderly persons are placed under general anesthesia^55^. In addition, general anesthesia use and incidence of dementia or cognitive imparement are positively correlated^56–58^. The risk of postoperative delirium is also high in elderly patients over 70 years of age or in patients with underlying intellecture disabilities^59^ which can lead to long-term cognitive imparement^60^. In these patients, performing therapeutic or tectonic keratoplasty as a preliminary step for future optical keratoplasty is not desirable due to the risk of systemic and neurologic complications related to anesthesia. Therefore, a method of obtaining therapeutic effects with a single operation, rather than repetitive operations, is clinically desirable. The therapeutic goal in emergency corneal transplantation is to maintain anatomical structure, eliminate infection, and, if possible, improve visual acuity. In the present study, patients who underwent tectonic keratoplasty exhibited significantly improved UCVA postoperatively relative to preoperative UCVA. In the therapeutic keratoplasty cohort, although there was no improvement in visual acuity, anatomical success was attained in 14 of 18 patients (77.8%). Because the main purpose of therapeutic keratoplasty is eradication of infected corneal tissue^50,54^, it could be reasonable to perform primary corneal transplantation using cryopreserved cornea tissue. Using this method, we can expect to preserve ocular integrity and improve visual acuity in non-infectious keratopathy, and to eliminate infected tissue in infectious keratitis, especially in elderly patients and/or patients with poor general condition.

There are several limitations to this study that should be acknowledged to avoid its overinterpretation. This study is a retrospective analysis, which is subject to selection bias. This is a case series study evaluating the advantage of using frozen cornea grafts for emergent corneal grafting situations in locations that lack donor corneas. However, the subjects of the study were heterogeneous and had various indications; therefore, a single study of a specific group controlled for age, race, and indication should be performed in the future. Further, there was no control group in the study. However, although direct comparison was limited, the results of the present study showed superior results compared to those of previous studies. Thanathanee et al.^29^ reported that among 22 patients that underwent therapeutic keratoplasty using glycerol-preserved corneas, nine patients (40.9%) received enucleation or evisceration, and 13 globes (59.1%) were preserved. In the present study, 4 of 18 patients (22.2%) underwent evisceration or enucleation, and ocular integrity was maintained in 14 patients (77.8%). Additionally, according to a study by Pant et al.^61^, who performed tectonic keratoplasty using a small incision refractive lenticule extraction-extracted lenticule, globe integrity was maintained in 16 of 18 patients (88.9%). In this study, ocular integrity was maintained in 27 (96.4%) of 28 patients that underwent tectonic keratoplasty. Nevertheless, it is necessary to compare keratoplasty using cryopreserved cornea with other surgical methods in a follow-up study. Further, the study relied on electronic medical records, and in several patients that underwent tectonic keratoplasty, the cause of perforation was not clearly identified, and the follow-up period was variable between patients. Patients with no final follow-up and patients under follow-up were included in analyses. Among patients with follow-up loss, a significant number of patients died due to underlying medical conditions early in the follow-up period. Patient 1 died from cerebral infarction, and Patient 31 died from subarachnoid hemorrhage during follow-up. Finally, only 19 of 46 patients were followed up for more than 1 year, which is insufficient to determine long-term surgical success. Although the study has the advantage of a relatively large number of cases with 55 cases in 46 patients, there were not enough cases to perform more subgroup analyses, for example ALK and PKP. And also the information collection period was relatively long at 11 years. The overall surgical procedure was the same, but there could have been changes in minor details over time. In this study, small diameter grafts or patterned grafts using PKP remnants were performed for several lesions. Therefore, the visual axis could have been affected or astigmatism could have occurred. However, there was insufficient information to determine the degree of astigmatism before and after surgery due to the nature of this study, in which emergency surgery was performed to treat corneal lesions (Table 5). In this study, frozen corneal grafts were transplanted for emergent surgeries. Early in the patient inclusion timeframe, DSAEK was not widely performed in Korea, so the number of frozen DSAEK lenticules was insufficient. Therefore, small grafts or patched grafts were performed despite the risk of astigmatism. Also, empirically, when a large diameter round graft was performed using residual anterior stromal lenticule after DSAEK in the same patient as case 5, this method led to bullous keratopathy because endothelial cells were not present, causing continuing pain in the patient and corneal vascularization. As large diameter round graft has some caveats, we determined that it was more appropriate to perform a graft as suitable as possible to the size of the lesion, and allow the endothelium of the recipient cornea to function. Next, it was not confirmed whether the antimicrobial effect of Optisol-GS remained after the corneal tissue was thawed before surgery, or whether infection by other bacteria or fungi occurred in the media. To prevent contamination, before the step of cryopreserving remnant donor cornea, fresh donor cornea was excised rapidly, such that contamination would not occur. Subsequently, the remnant tissue was immediately and aseptically immersed in Optisol-GS and cryopreserved. However, a prior study suggested that the antimicrobial effect of Optisol-GS is diminished at low temperatures^38^. If the storage period is prolonged, the antimicrobial effect could decrease or infection of the media could occur, affecting postoperative outcomes. Finally, there is currently no legislation in Korea restricting the transplantation of the same organ to multiple recipients. Therefore, this procedure may not be possible in countries where it is prohibited to transplant the same cornea more than once. Further prospective and long-term follow-up study of a larger number of patients is needed to validate our findings.

In conclusion, although comparisons with control groups are necessary, therapeutic or tectonic keratoplasty using cornea cryopreserved in Optisol-GS is potentially an inexpensive, safe, and effective surgical option that can be expected to maintain anatomical integrity in cases of corneal perforation and infectious keratitis. Additionally, improved visual acuity can be expected in patients who undergo tectonic keratoplasty using cryopreserved cornea. In elderly patients or patients with poor general condition, who are highly likely to undergo the above procedures, use of cryopreserved corneal tissue can be considered a superior method to keratoplasty using fresh donor cornea. This method can reduce side effects by reducing the use of immunosuppressants, and successful treatment can be obtained with a single procedure without further surgeries in most cases.

## Supplementary Information

Supplementary Information.

## Data Availability

The datasets generated and analyzed during the current study are available in a supplementary file.
